# Association between Swallowing Outcomes and Dose to Critical Swallow Structures in Patients Undergoing Transoral Robotic Surgery and Post-Operative Radiation Therapy

**DOI:** 10.1007/s00455-024-10719-w

**Published:** 2024-06-05

**Authors:** Emma Charters, Anna Lawless, Jonathan R Clark, Natalie McCabe, Chris Milross, Rafe Britton, Gillian Heller, Raymond Wu

**Affiliations:** 1 Department of Head and Neck Surgery, Chris O’Brien Lifehouse, 119-143 Missenden Road, Camperdown, Sydney, NSW 2050 Australia; 2 School of Health Sciences, Faculty of Medicine and Health, The University of Sydney, Sydney, Australia; 3 Sydney Medical School, Faculty of Medicine and Health Sciences, The University of Sydney, Sydney, Australia; 4 Department of Radiation Oncology, Chris O’Brien Lifehouse, Sydney, Australia; 5 Royal Prince Alfred Institute of Academic Surgery, Sydney Local Health District, Sydney, Australia; 6 Lubrication Expert, Sydney, Australia

**Keywords:** Oral neoplasm, Dysphagia, Robotic surgery, Dysphagia and aspiration related structures

## Abstract

**Background:**

The radiation dose to dysphagia and aspiration-related structures (DARS) for patients undergoing transoral robotic surgery (TORS) and post-operative radiation therapy (PORT) for primary oropharyngeal carcinoma is unknown.

**Methods:**

This prospective study measured swallowing using the MD Anderson Dysphagia Inventory at baseline and then 12-months after PORT. Dosimetric parameters were collected.

**Results:**

19 patients were recruited between 2017 and 2019. Worse swallow function at 12-months after PORT was associated with dose-parameters to the oesophageal inlet muscle, superior pharyngeal constrictor muscle and cervical oesophagus. Mean dose, V50Gy, and V60Gy to the base of tongue and pharyngeal constrictors was significantly lower in those receiving PORT to the neck alone.

**Conclusion:**

Dose to DARS was lower in patients who received PORT to the neck alone. In patients treated with TORS and PORT, poorer swallowing outcomes at 12 months post-treatment were associated with increased dose to oesophageal inlet muscle, superior constrictor muscle, and cervical oesophagus.

## Introduction

Primary transoral robotic surgery (TORS) and definitive intensity modulated radiation therapy (IMRT) are both accepted treatment options for early-stage oropharyngeal cancer (OPC) with excellent survival outcomes and similar quality of life [1, 2]. The decision regarding treatment modality is based on individual patient and tumour factors, with the aim of optimising functional outcomes, in particular speech and swallow, without compromising oncological outcomes. Upfront TORS allows unimodality treatment in appropriately selected patients [3, 4] and pathologically directed adjuvant treatment in others, including de-intensification of radiation therapy (RT) and/or omission of chemotherapy [2, 5–7].

A randomised trial comparing definitive RT and TORS for OPC is inherently difficult. The ORATOR trial demonstrated that primary RT was associated with superior swallowing function compared to TORS at 1, 2 and 3 years. However, the difference was statistically significant only at 1 year, and did not meet the threshold for a clinically meaningful change at any timepoint [1]. Various limitations of the ORATOR trial have been cited: small sample size, ‘over-treatment’ with adjuvant therapy, larger than necessary surgical margins, and the overuse of elective tracheostomies. The ORATOR2 trial attempted to evaluate de-escalated treatment of HPV-related OPC, by comparing surgical treatment (TORS and neck dissection followed by adjuvant RT based on pathology) versus non-surgical treatment (primary RT to 60 Gy with or without chemotherapy) [8]. Swallow outcomes at 1 year were very good in both arms, however the trial was closed early due to two treatment-related deaths in the surgical arm. Retrospective studies suggest dysphagia at six months after completion of treatment is similar for patients who undergo primary TORS and neck dissection versus non-surgical management of early OPC [9].

Recently-published randomised data supports the benefit of dysphagia-optimised intensity-modulated radiotherapy (DO-IMRT) for patients with oropharyngeal or hypopharyngeal cancer undergoing bilateral neck irradiation. A significant reduction in mean dose to the pharyngeal constrictor muscles was achieved using DO-IMRT, which correlated with improved swallow related quality of life at 12 months [10]. Omission of RT to the primary site following TORS is another method to reduce treatment related toxicities and improve swallow function and quality of life. Various studies suggest omission of adjuvant irradiation of the primary site in well-selected patients may result in improved swallowing and speech outcomes while maintaining excellent local control [2, 3, 11, 12]. For patients undergoing definitive RT, increased dose received by dysphagia and aspiration-related structures (DARS) is associated with worse physical, functional and patient reported outcomes [13–20]. This study aimed to describe the relationship between dose to DARS and swallow outcomes for patients undergoing TORS followed by PORT for early stage OPC.

## Methods

### Study Design and Participants

A prospective, non-randomized cohort study was undertaken in a single Australian tertiary oncology centre. Recruitment was carried out consecutively between January 2017 and May 2019. The trial was approved by the Royal Prince Alfred Research and Ethics committee (Sydney, Australia; Protocol X17-0047). All participants provided written informed consent.

Eligible patients were aged 18 years or over with AJCC 8th edition clinical stage I (T1-2 N0-1 M0) histologically-confirmed squamous cell carcinoma of the oropharynx, who were suitable for primary TORS and unilateral neck dissection and received post-operative radiation therapy (PORT) at our institution. Any p16/HPV status were eligible for inclusion. All cases were discussed in a dedicated multidisciplinary team (MDT) meeting prior to treatment. Patients deemed suitable by the MDT for either primary TORS or RT discussed these options with a surgeon and radiation oncologist before deciding on and proceeding with treatment. Patients were not eligible if they had a pre-existing diagnosis which might contribute to a communication or swallowing impairment or underwent TORS as a salvage procedure.

### Treatment

Patients were considered suitable for single-staged TORS and unilateral selective neck dissection for cT1-2 N0-1 tonsil or lateralized base of tongue SCC, where clear margins could be reasonably achieved with favourable morbidity based on clinical and radiological assessment. In the case of close surgical margins (< 1 mm), re-resection was recommended where possible, prior to consideration of PORT.

PORT to the ipsilateral neck was recommended for patients with pathological evidence of two or more lymph nodes, or presence of extranodal extension (ENE), and considered for a single node > 3 cm without ENE. PORT to the primary tumour bed was recommended for positive margins (where re-resection was not possible or would result in an unacceptable functional outcome), or presence of multiple adverse features including perineural invasion (PNI), lymphovascular invasion (LVI), close margins (< 1 mm), poorly differentiated tumour, or endophytic growth pattern with infiltrating borders. Our institution has historically had a preference towards de-escalating adjuvant radiotherapy to the primary site following TORS, and has previously reported on excellent rates of local control with this approach [3]. Concurrent chemotherapy with PORT was recommended for positive margins, ENE, or extensive nodal burden.

PORT was delivered to a dose of 60 Gy in 2 Gy per fraction over 6 weeks, with an optional simultaneous integrated boost to 63–66 Gy to areas of ENE or microscopic positive margins. Patients deemed to have low-moderate risk were treated with low-intermediate risk disease were treated to 54 Gy (1.8 Gy per fraction) at one of the treating clinicians’ discretion, similar to the approach tested in the ECOG-ACRIN E3311 trial [5]. All patients were treated using intensity-modulated radiotherapy (IMRT) or RapidArc (VMAT) technique, with custom thermoplastic mask immobilisation and daily image guidance. Treatment plans were optimised according to the standard departmental protocol, without employing a ‘dysphagia-optimised IMRT’ technique such as used in the recently published ‘DARS’ study [10]. DARS that were not contoured at the time of initial treatment were retrospectively contoured (according to guidelines by Christianen et al.) by a clinician blinded to the dose distribution, for inclusion in the dosimetric analysis [21].

### Dosimetric Analysis

DARS included the superior, middle and inferior constrictor muscles (SCM, MCM, ICM respectively); cricopharyngeus muscle (CPM); oesophageal inlet muscle (OIM); cervical oesophagus (CE); base of tongue (BOT); supraglottic larynx (SL) and glottic larynx (GL) [21]. Dosimetric parameters were extracted from the treatment planning system (Eclipse, Varian Medical Systems, Inc), including the mean dose, V50Gy, and V60Gy (the percentage of structure receiving at least 50 Gy or at least 60 Gy respectively).

### Swallowing

Patient and clinician reported outcomes were prospectively recorded prior to TORS, and then 12 months post-completion of RT. All participants completed the MD Anderson Dysphagia Inventory (MDADI) [22], a 20-item instrument using a 5-point Likert scale to assess global, physical, emotional, and functional swallowing symptomology. Higher MDADI scores (up to total composite score of 100) indicate superior functioning and higher swallow-related quality of life.

Our institution places a nasogastric tube (NGT) inserted at the time of TORS. Patients commence oral trials with the Speech Pathologist between day 1 and 3 post-operatively, starting with water then upgrading texture based on surgical clearance. The NGT is removed after the surgical, nursing, and allied health teams came to a consensus that the patient has demonstrated their ability to manage an oral diet, at which time the tube would be removed. All participants were reviewed during their inpatient surgical admission then weekly during radiation therapy by the treating Radiation Oncologist, dedicated Nurse Practitioner, Dietitian and Speech Pathologist, with standard proactive symptom management of acute toxicities. Routine swallow interventions included compensatory and rehabilitation exercises specific to the participant’s presenting condition as per usual clinical practice, although there was no standardised protocol for assessment and rehabilitation of swallow function as part of this study. Participants were encouraged to continue oral intake during PORT as a proactive approach to swallow therapy [23].

### Statistical Analysis

Statistical analysis was carried out with system software R i386 3.2.2 (The R Foundation for Statistical Computing). Generalized Estimating Equations (GEE) were used to model the trajectory of swallowing outcome scores from pre-surgery to 12-months post-radiotherapy using the *geepack* and *lme4* packages for each individual clinical and dosimetric variable. GEEs allow incorporation of participants with missing data at selected time points. The beta score is the coefficient of each predictor, arising out of the linear model for the MDADI score at 12 months. The confidence intervals (CIs) were calculated to reflect the corresponding 95% CIs for beta.

## Results

### Participants

There were 44 patients who underwent TORS for early stage OPC between 2017 and 2019. Of these, 25 patients were excluded for analysis for reasons including receiving PORT at an external site (*n* = 6), not receiving PORT (*n* = 18), or radiotherapy to the bilateral neck (*n* = 1). The remaining 19 patients underwent TORS + PORT (to ipsilateral neck +/- primary site) and were included in the final analysis. Patient demographics, tumour stage and subsite, and treatment details are listed in Table 1. Primary tumours arose from the tonsil in 12 patients (63%), and base of tongue in seven patients (37%). The majority of tumours were T2 (*n* = 12, 63%) and node positive (*n* = 18, 95%). Almost all tumours were p16 positive (*n* = 18, 95%). No patients experienced disease relapse or required further treatment during the study follow-up period.

**Table 1:** Subject demographics and treatment details

	TORS + PORT (*n* = 19)	TORS + PORT (*n* = 19)	
		RT primary & neck (*n* = 5)	RT neck only (*n* = 14)
Age (years) (mean, range)	57.2 (47–71)	64.7 (55–75)	61.5 (53–72)
Gender			
Female	3 (15.8%)	2 (40.0%)	1 (7.1%)
Male	16 (84.2%)	3 (60.0%)	13 (92.9%)
Primary tumour location			
Base of tongue	7 (36.8%)	2 (40.0%)	5 (35.7%)
Tonsil	12 (63.1%)	3 (60.0%)	9 (64.3%)
T-Category			
1	7 (36.8%)	3 (60.0%)	4 (28.6%)
2	12 (63.1%)	2 (40.0%)	10 (71.4%)
N-Category			
0	1 (5.2%)	1 (20.0%)	0 (0.0%)
1	14 (73.7%)	1 (20.0%)	13 (92.9%)
2	4 (21.0%)	3 (60.0%)	1 (7.1%)
p16 status			
Positive	18 (94.7%)	4 (80.0%)	14 (100.0%)
Negative	1 (5.3%)	1 (20.0%)	0 (0.0%)
Smoking status			
Never	13 (68.4%)	2 (40.0%)	11 (78.6%)
Ex-smoker	6 (31.6%)	3 (60.0%)	3 (21.4%)
Current	0 (0.0%)	0 (0.0%)	0 (0.0%)
RT Dose (Gy / fraction)			
54/30	5 (26.3%)	0 (0.0%)	5 (35.7%)
60/30	10 (52.6%)	3 (60.0%)	7 (50.0%)
63/30	3 (15.8%)	1 (20.0%)	2 (14.3%)
66/30	1 (5.3%)	1 (20.0%)	0 (0.0%)
Concurrent systemic therapy	6 (31.6%)	3 (60.0%)	3 (21.4%)
Cisplatin	3 (15.8%)	2 (40.0%)	1 (7.1%)
Carboplatin	2 (10.2%)	0 (0.0%)	2 (14.3%)
Cetuximab	1 (5.3%)	1 (20.0%)	0 (0.0%)
None	13 (68.4%)	2 (40.0%)	11 (78.6%)

Values are Displayed as mean (range), or total (%)

American Joint Committee on Cancer (AJCC), Tumour (T), Node (N), Chemo-radiation (CRT), Transoral Robotic Surgery (TORS), post-operative radiation therapy (PORT), radiation therapy (RT), Radiation Gray (Gy)

*AJCC 8th ed. pathologic T- & N-category for TORS + PORT, clinical T- & N-category for definitive CRT.

### Radiation Therapy

Of the 19 patients in the TORS + PORT group, the majority (*n* = 14, 74%) received PORT to the ipsilateral neck alone, and the remainder (*n* = 5, 26%) received treatment to the primary site and ipsilateral neck. Median and most commonly prescribed dose was 60 Gy in 30 fractions (range 54–66 Gy in 30 fractions). The most common indication for PORT was nodal involvement and extranodal extension. All patients completed treatment in full. Six patients (32%) received concurrent chemotherapy (Table 1).

Mean dose, V50Gy, and V60Gy to the base of tongue, superior, and middle pharyngeal constrictor was significantly lower in those who received PORT to the neck alone, compared to patients where the primary site was treated (Table 2).

**Table 2:** Dosimetric data for critical dysphagia and aspiration related structures (DARS)

Structure	RT	Mean dose (Gy) Mean (SD)	*p*	V50Gy (%) Median (IQR)	*p*	V60Gy (%) Median (IQR)	*p*
Base of tongue	All	34.9 (10.0)		6.0 (2.7–29.5)		0.0 (0.0–14.0)	
	Primary + neck	43.1 (9.5)	**0.01**	34.6 (18.0-65.7)	**0.01**	18.1 (6.9–38.9)	**0.02**
	Neck	32.2 (8.7)		3.4 (1.3-9.0)		0.0 (0.0-0.01)	
Superior pharyngeal constrictor	All	34.8 (11.6)		19.2 (5.9–45.1)		0.5 (0.0-23.4)	
	Primary + neck	47.0 (7.3)	**0.01**	54.8 (28.4–75.2)	**0.006**	37.1 (9.8–44.0)	**0.007**
	Neck	30.8 (9.8)		14.6 (4.9–22.5)		0.0 (0.0-5.7)	
Middle pharyngeal constrictor	All	40.3 (6.3)		27.0 (10.3–36.4)		1.6 (0.0-11.8)	
	Primary + neck	44.5 (6.9)	**0.02**	38.1 (32.0-67.8)	**0.07**	16.3 (5.1–22.5)	**0.01**
	Neck	38.9 (5.5)		20.5 (6.8–28.5)		0.0 (0.0-6.3)	
Inferior pharyngeal constrictor	All	34.3 (7.4)		7.8 (1.6–19.5)		0.0 (0.0-0.02)	
	Primary + neck	38.0 (7.2)	0.8	19.1 (8.9–27.1)	0.6	0.0 (0.0–0.0)	0.9
	Neck	33.0 (7.2)		4.8 (0.0-15.6)		0.0 (0.0-0.1)	
Crico-pharyngeus muscle	All	34.6 (6.4)		5.0 (2.3–15.9)		0.0 (0.0–0.0)	
	Primary + neck	35.3 (8.4)	0.7	10.7 (0.0-1.9)	0.08	0.0 (0.0-0.2)	0.3
	Neck	34.4 (5.9)		4.5 (0.0-16.6)		0.0 (0.0–0.0)	
Oesophageal inlet muscle	All	36.5 (5.4)		0.9 (0.0-9.1)		0.0 (0.0–0.0)	
	Primary + neck	35.3 (6.3)	0.5	0.00 (0.0–0.0)	0.3	0.0 (0.0–0.0)	
	Neck	37.2 (5.2)		2.7 (0.0-11.2)		0.0 (0.0–0.0)	
Cervical oesophagus	All	30.0 (7.1)		0.00 (0.0-5.2)		0.0 (0.0–0.0)	
	Primary + neck	27.9 (7.8)	0.7	0.0 (0.0–0.0)	0.9	0.0 (0.0–0.0)	
	Neck	30.6 (7.0)		1.4 (0.0-11.2)		0.0 (0.0–0.0)	
Supraglottic larynx	All	35.6 (8.5)		8.4 (4.4–19.3)		0.0 (0.0-0.2)	
	Primary + neck	38.2 (12.5)	0.9	25.2 (6.3–52.4)	0.09	0.0 (0.0-24.8)	0.3
	Neck	34.8 (7.1)		8.1 (1.8–11.6)		0.0 (0.0-0.2)	
Glottic larynx	All	29.5 (8.3)		1.3 (0.02–5.8)		0.0 (0.0–0.0)	
	Primary + neck	33.5 (8.4)	0.5	2.9 (0.1–28.6)	0.3	0.0 (0.0-0.6)	
	Neck	28.2 (8.1)		1.0 (0.0–5.0)		0.0 (0.0–0.0)	

p values reflect difference in scores between the two adjuvant regimens (primary + neck vs. neck only)

Primary + neck: transoral robotic surgery (TORS) + adjuvant radiotherapy to primary site and ipsilateral neck

Neck: TORS + adjuvant radiotherapy to ipsilateral neck

### Swallowing

Following TORS, 17 (89%) participants received nasogastric tube (NGT) feeding (mean 6 days, range 0–24 days). All participants were consuming an oral diet prior to commencing PORT. Two patients (10%) who underwent definitive RT had a prophylactic gastrostomy tube placed prior to treatment, and one had a reactive NGT. These were removed by 6 months post treatment.

**Fig. 1:**
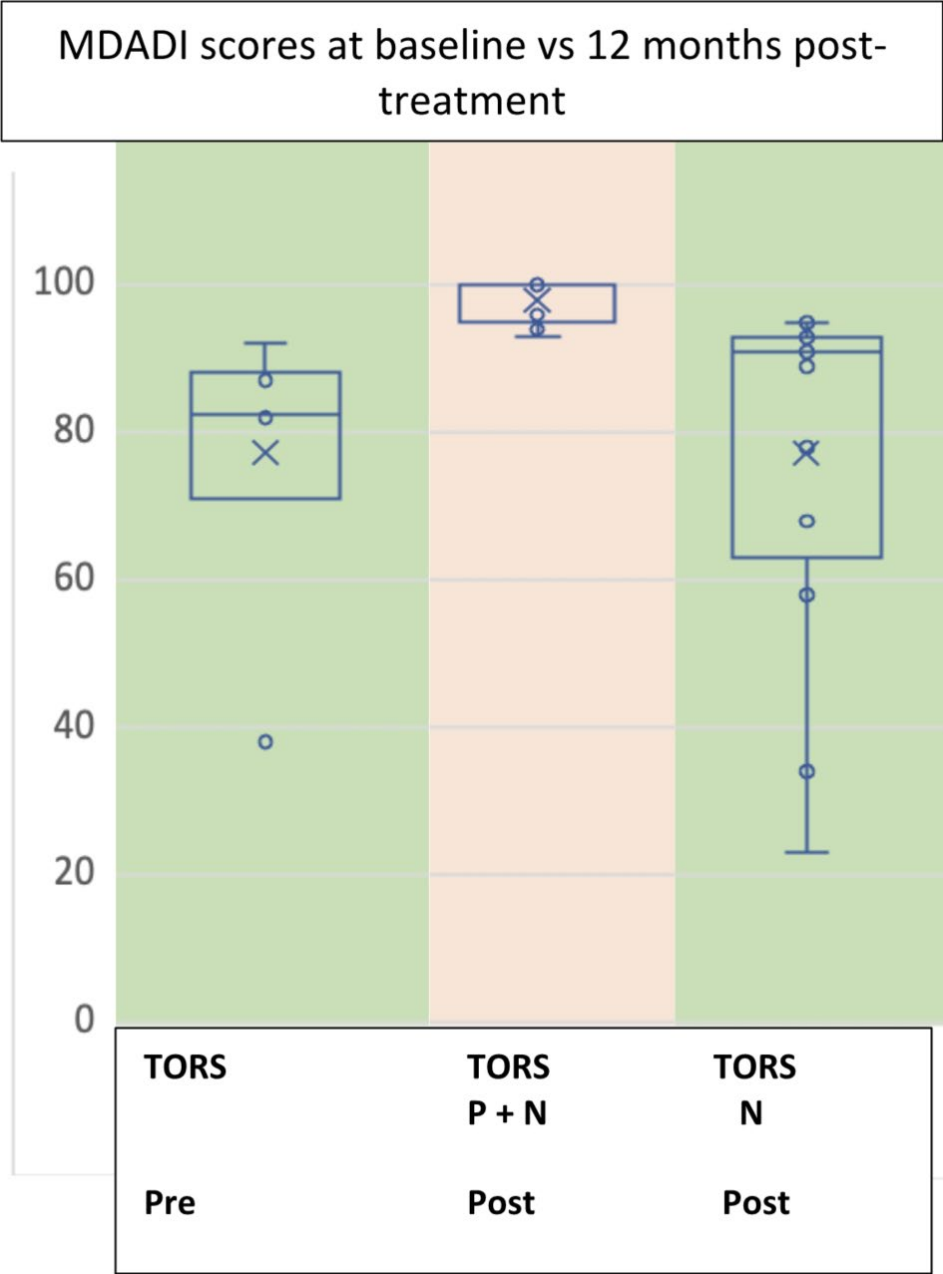
MDADI scores for all patients according to treatment group between baseline (pre-treatment) and 12-months post-treatment. TORS Pre: Baseline transoral robotic surgery with adjuvant radiation. TORS P + N Post: 12 months post transoral robotic surgery with adjuvant radiation to the primary site and neck, TORS N Post: 12 months post transoral robotic surgery with adjuvant radiation to the neck only

Median MDADI score at baseline prior to TORS was 100 (range 95–100). At 12 months post-treatment, median MDADI score was higher in the patients who had PORT with omission of the primary site (median 91, range 23–95) compared to those where the primary site was included (median 83, range 38–92) (Fig. 1).

### Relationship between Dose to DARS and Swallow Outcomes

At 12 months post RT, poorer swallowing outcomes (lower MDADI) were associated with dose-parameters to OIM (V50Gy and V60Gy, beta − 1.7, *p* < 0.001 and beta − 1.5, *p* = 0.008, respectively), SPCM (V60Gy, beta − 0.98, *p* = 0.04) and CE (V60Gy, beta − 0.56 *p* = 0.05) (Table 3). Addition of chemotherapy was also associated with lower MDADI at 12 months (beta − 7.9, *p* = 0.05).

**Table 3:** Association between clinical and dosimetric parameters and MDADI score at 12 months

Characteristic	Beta	95% CI	*p*-value
Baseline MDADI	3.0	-1.2, 7.1	0.2
**Chemotherapy (Y)**	**-7.9**	**-9.2, -5.7**	**0.05**
**SPCM V60Gy**	**-0.94**	**-0.46, 0.98**	**0.05**
**OIM V50Gy**	**-1.7**	**-2.4, -1.0**	**< 0.001**
**OIM V60Gy**	**-1.5**	**-1.9, -1.2**	**0.008**
**CE V60Gy**	**-0.56**	**-1.3, 0.2**	**0.05**

Bolded variables indicate a significant relationship with worse swallow function (lower MDADI score)

V50/60Gy = Volume of structure receiving at least 50/60Gy

Superior constrictor muscle (SPCM), oesophageal inlet muscle (OIM), Cervical oesophageous (CE), MD Anderson Dysphagia Inventory (MDADI).

## Discussion

This study is the first to report the relationship between dose to DARS and clinical swallow outcomes in the adjuvant post-TORS setting. The vast majority of published literature regarding the relationship between dosimetric parameters during head and neck radiotherapy and swallowing function exists in the setting of definitive radiotherapy. This study quantifies the radiation dose to DARS in a cohort of 19 patients undergoing TORS and PORT to a unilateral volume. Inclusion of the primary site in PORT was unsurprisingly associated with higher dose to DARS in close proximity to the oropharynx (BOT, SPCM, MPCM). Worse swallow function at 12 months post-treatment was associated with increased dose to OIM, SPCM and CE. These findings align with previous literature in the definitive RT setting, where increased dose to the pharyngeal constrictors and oesophagus is associated with worse dysphagia [21, 24–26], and suggest possible areas for refinement of dysphagia-optimised IMRT (DO-IMRT) in the post-TORS setting.

Proponents of TORS for early-stage OPC emphasise the advantage of decreased RT dose to swallowing structures [27–29]. As expected, this study demonstrated consistently lower dose to DARS (specifically dose to SPCM, MPCM and BOT) where RT to the primary site was omitted. Most notably, mean dose to SPCM (31.3 Gy vs. 43.4 Gy), and to BOT (31.3 Gy vs. 43.6 Gy) was significantly lower for RT to neck alone vs. primary + neck. Even larger differences were seen in V50Gy and V60Gy, dose levels traditionally thought to be most strongly correlated with long-term dysphagia and were used as planning aims in the recently published DARS study (mean < 50 Gy) [10]. Acknowledging that the sample size for each subset were low for the number of variables analysed, we observed higher MDADI scores for patients where radiotherapy to the primary site was omitted (median 91 vs. 82.5). Future studies should explore this relationship with appropriately powered participant numbers. There is growing evidence from the AVOID trial and single institution series to show that treatment de-escalation by selectively omitting primary site irradiation is able to safely preserve excellent oncologic outcomes while optimizing functional outcomes.

In this cohort of early stage OPC receiving unilateral treatment, the high dose volumes to the oesophageal inlet muscle, superior constrictor muscle and cervical oesophagus were the only DARS dosimetric parameters that were associated with poorer swallow function at 12 months. This suggests a possible benefit to prioritizing an optimization objective of reduced 50–60 Gy to these structures, however external validation from larger data sets is required before translating these findings into clinical practice. It is also possible that the current study’s small sample size and relatively homogeneous doses to other swallowing structures may have hindered the ability to detect of other associations between DARS dosimetry and swallowing dysfunction that may exist.

This study adds to the extensive literature on swallow outcomes after treatment for OPC by reporting detailed dosimetric data alongside swallowing outcome measurements at 12 months. Firstly, the dose de-escalation achieved by a TORS and pathologically guided adjuvant RT is quantified, specifically the impact of omission of RT to the primary site. Secondly, this study observed high dose volumes (V50Gy, V60Gy) to OIM, SPCM and CE were associated with worse swallow outcomes, generating the hypothesis that optimizing for these during the planning process may reduce treatment-related dysphagia. Incremental improvements in swallow related quality of life remain an important priority in a cohort of patients with excellent survival outcomes.

This study was conducted in a single institution, which may skew the results due to institution-specific treatment preferences (e.g., a willingness to omit the primary site from PORT volume) and limit the generalizability of results. The study analysed a relatively small population, leading to reduced statistical power to detect statistically significant associations between dosimetry and swallowing, and biased estimates due to the influence of outliers. Despite the use of the validated MDADI tool, the lack of instrumental swallow assessment (i.e., fibreoptic endoscopic swallow study or videofluoroscopic swallow study) means the physiological impact of RT de-escalation on oropharyngeal function cannot be commented upon. Although the 12-month quality of life follow-up duration is useful to assess medium term toxicities of treatment, longer term follow up is also required given the excellent prognosis in this population. When considering larger, prospective studies for the future, employing a minimally clinically important difference for the MDADI (identified as a 10-point between-group difference in the composite score), would add to the clinical relevance of the outcomes[28]. Despite these limitations, this prospective cohort study quantifies radiation dose to DARS in patients undergoing TORS and PORT and provides early evidence to inform optimization of RT treatment protocols in the post-TORS setting.

## Conclusion

In patients undergoing PORT after TORS, higher radiation dose to the superior pharyngeal constrictor muscle (V60Gy), oesophageal inlet muscle (V50Gy and V60Gy), and cervical oesophagus (V60Gy) were predictive of worse swallowing outcomes at 12 months. We observed lower doses to certain DARS (base of tongue, superior and middle pharyngeal constrictors) and improvements in post-treatment swallow function in patients receiving PORT to the neck alone (versus primary + neck), suggesting that further study is warranted to assess the effectiveness and safety of this approach as a method of treatment de-escalation.

## Data Availability

The data that support the findings of this study are available from the authors upon request.
